# Indications for 3-D diagnostics and navigation in dental implantology with the focus on radiation exposure: a systematic review

**DOI:** 10.1186/s40729-021-00328-9

**Published:** 2021-05-27

**Authors:** Burkhard Kunzendorf, Hendrik Naujokat, Jörg Wiltfang

**Affiliations:** Department of Oral and Maxillofacial Surgery, University Hospital of Schleswig-Holstein, Campus Kiel, Arnold-Heller-Straße 3, 24105 Kiel, Germany

**Keywords:** Dental implants, Cone beam CT, Navigation, 3-D imaging, 2-D imaging

## Abstract

**Background:**

Dental implants are a common restorative method used to replace missing teeth. Implant placement techniques guided by three-dimensional imaging and navigation are becoming more widely available.

**Objective:**

The present review focused on the following questions: 1. What are the advantages and disadvantages of 2-D versus 3-D imaging in dental implantology? 2. What are the advantages and disadvantages of freehand implant placement in comparison with navigation-guided implant placement?

**Methods:**

A systematic review was performed, based on the Preferred Reporting Items for Systematic Reviews and Meta-analysis (PRISMA) statement. The following libraries were searched for relevant literature: PubMed, Embase, Arbeitsgemeinschaft der Wissenschaftlichen Medizinischen Fachgesellschaften (AWMF) Online, and the Cochrane Library. The risk of bias was assessed using the Scottish Intercollegiate Guidelines Network (SiGN) checklist. A total of 70 studies were included after screening, and the evidence from these was gathered for review.

**Results:**

Three-dimensional imaging is advantageous in terms of image quality, and it provides a distortion-free evaluation of the implant site. However, it is also associated with higher costs and increased radiation exposure. Dynamic and static navigation are equal in accuracy and are both more accurate compared with the freehand method. No benefit in terms of implant survival could be demonstrated within the first 5 years for any specific method.

**Discussion:**

A panoramic X-ray with a reference body often provides sufficient imaging and is the primary method for two-dimensional imaging. Cone beam computed tomography with low-dose protocol settings should be used if three-dimensional imaging is needed. Navigational support should be considered in the event of especially complex cases.

**Conclusion:**

The guidance technique used for implant placement should be decided on an individual basis. With the increasing availability of three-dimensional imaging, there should also be an increase in awareness of radiation exposure.

**Supplementary Information:**

The online version contains supplementary material available at 10.1186/s40729-021-00328-9.

## Background

Dental implants are a well-established method of prosthetic oral rehabilitation. High-quality imaging of the bone and surrounding anatomical structures is necessary for proper diagnosis, implant-planning, and implant placement. In many cases, two-dimensional (2-D) radiographic images are sufficient; however, if all relevant anatomical structures cannot be evaluated, or if further information is needed [1, 2], three-dimensional (3-D) imaging might be helpful [3].

Throughout their history, many dental implants were placed using the freehand method. Although an experienced surgeon can achieve good results with this method, the use of static or dynamic navigation is well established and seems to improve the outcome in terms of placement accuracy, while protecting vulnerable adjacent structures [4].

The present review was conducted to evaluate the advantages and disadvantages of 2-D versus 3-D imaging techniques, as well as those of different navigation methods. This review aimed to provide a more detailed view of guidance techniques for implant placement.

## Materials and methods

The present systematic review was based on the Preferred Reporting Items for Systematic Reviews and Meta-analysis (PRISMA) statement, with two focused research questions:

1. What are the advantages and disadvantages of 2-D versus 3-D imaging techniques in dental implantology?
2. What are the advantages and disadvantages of freehand implant placement in comparison with navigation-guided implant placement?

### Search strategies

The systematic literature search for the present review was performed using the following databases: PubMed, Embase, Arbeitsgemeinschaft der Wissenschaftlichen Medizinischen Fachgesellschaften (AWMF) Online, and the Cochrane Library. Literature published from 2010 to September 2019 was searched for eligible articles. Endnote X9 was used as the citation software. The search criteria were based on two population, intervention, comparison, outcome (PICO) questions using the following terms:

1. **P**opulation: dental implant

**I**ntervention: cone beam computed tomography (CBCT), cone beam CT, multi-slice computed tomography (MSCT), multi-slice CT

**C**omparison: orthopantomogram, panoramic X-ray, pantomogram, dental panoramic radiograph

**O**utcome: bone quality, distance measurement, inferior alveolar nerve, incisive nerve, radiation, guideline, review, cost, outcome, resolution, accuracy

2. **P**opulation: dental implant, endosseous implant

**I**ntervention: guided, navigation (static, dynamic)

**C**omparison: freehand

**O**utcome: accuracy, survival, failure, nerve, peri-implantitis, pain, positioning, cleft

The search terms for the two PICO questions lead to a variety of combinations. The following list presents the search combinations as it was used for the main source (PubMed):

1. PICO:

1. CBCT; 2. cone beam CT; 3. orthopantomogram; 4. panoramic X-ray; 5. pantomogram; 6. dental panoramic radiograph: 7. MSCT; 8. multi-slice CT; 9. dental implant; 10. bone quality; 11. distance measurement, 12. inferior alveolar nerve; 13. incisive nerve; 14. radiation; 15. guideline; 16. review; 17. cost; 18. outcome; 19. resolution; 20. accuracy

**Table:** 

Search combinations	Search results	Title /abstract	Full text	Included
(3 OR 4 OR 5 OR 6) AND (1 OR 2 OR 7 OR 8) AND 10	13	7	4	1
(3 OR 4 OR 5 OR 6) AND (1 OR 2 OR 7 OR 8) AND 11	8	7	5	3
(3 OR 4 OR 5 OR 6) AND (1 OR 2 OR 7 OR 8) AND 12 OR 13 AND 9	48	11	9	7
(3 OR 4 OR 5 OR 6) AND (1 OR 2 OR 7 OR 8) AND 14	77	34	26	11
(3 OR 4 OR 5 OR 6) AND (1 OR 2 OR 7 OR 8) AND 17	18	16	1	0
(15 OR 16) AND 1 AND 9	86	47	24	15
1 AND 7 AND 19	29	13	10	7
1 AND 7 AND 20	42	23	18	12
(15 OR 16) AND 9				2
12 (freehand)				3

2. PICO:
  1. freehand; 2. guided; 3. navigation; 4. dental implant; 5. endosseous implant; 6. accuracy; 7. survival; 8. failure; 9. nerve; 10. peri-implantitis; 11. pain; 12. positioning; 13. dynamic navigation, 14. static navigation; 15. cleft

**Table:** 

Search combinations	Search results	Title/abstract	Full text	Included
1 AND 2 AND 4 AND 6	13	8	6	5
1 AND 2 AND 4 AND 7	4	4	3	1
1 AND 2 OR 3 AND 4 OR 5 AND 8	4	4	3	0
1 AND 2 OR 3 AND 4 OR 5 AND 9	1	1	1	0
1 AND 2 OR 3 AND 4 OR 5 AND 10	1	1	1	0
1 AND 2 OR 3 AND 4 OR 5 AND 11	2	2	2	2
1 AND 2 OR 3 AND 4 OR 5 AND 12	8	5	3	0
1 AND 2 OR 3 AND 4 OR 5 AND 15	0	0	0	0
13 AND 14 AND 4 AND 5	14	11	5	3
(4 OR 5) AND 1 AND 3	2	2	2	0

### Study inclusion and exclusion criteria

**Fig. 1:**
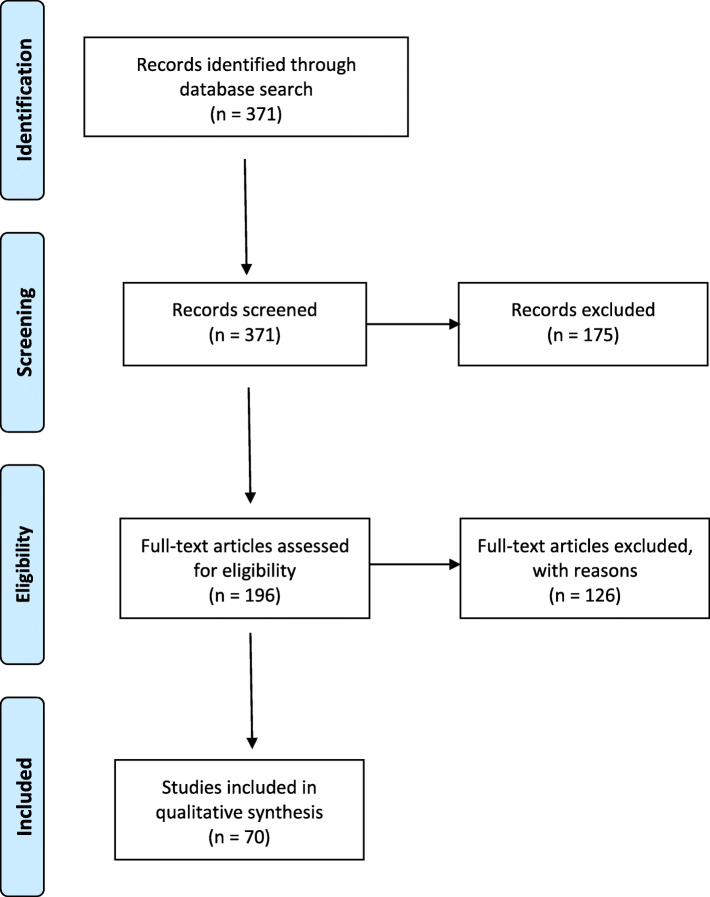
Study selection process Modified from auto-comp. Moher D, Liberati A, Tetzlaff J, Altman DG, The PRISMA Group (2009). Preferred Reporting Items for Systematic Reviews and Meta-Analyses: The PRISMA Statement. PLoS Med 6(7): e1000097. doi:10.1371/journal.pmed1000097

For study selection, the titles and abstracts were screened first, using the following inclusion criteria: English or German language and a clinical study. Due to the nature of comparative studies involving radiation, experimental studies were included in the search for the first PICO question. After the initial screening process, the studies were evaluated and excluded for the following reasons: studies older than 2010, case reports, and studies with less than 10 participants. The selection process is illustrated in Fig. 1.

### Risk of bias assessment for the selected studies

The risk of bias and the quality of the studies were assessed using the Scottish Intercollegiate Guidelines Network (SiGN) checklist.

## Results

### Study selection

Overall, the research questions led to a variety of subtopics. Sufficient quality and quantity of data were available; however, due to the heterogeneity of the material, conducting a meta-analysis was not feasible. The study types varied by topic, for example, studies on radiation exposure tended to be experimental with a highly consistent setup, implant accuracy, and survival were primarily investigated in retrospective analysis and summarized in reviews, although randomized controlled trials were available.

Overall, 4 guidelines, 12 systematic reviews, 15 narrative reviews, 8 randomized controlled trials, 1 prospective cohort study, 10 retrospective analyses, and 20 experimental studies were included in the present review, emphasizing the broad availability of guidelines and systematic reviews.

### Risk of bias

The risk of bias in the studies was assessed using the SiGN-checklist [5]. Overall, it appeared that there was a low risk of bias due to the high quality and the large number of studies available. The risk of bias for the included studies is listed in the attached evidence table (see Additional file 1).

### Indications for 3-D imaging

In order to safely and accurately place an implant, there must be, in addition to the relevant clinical information, a sufficient radiological image of the bone and adjacent tissue and anatomic structures [6, 7]. A basic principle, when using radiation for diagnostics, is that the lowest amount of radiation should be used to obtain the required information [8]. Usually, a 2-D image with a reference body, like a panoramic X-ray, provides sufficient information [2, 9, 10]. If 2-D imaging is not sufficient, 3-D imaging, such as CBCT is needed [11]. Occasionally, clinical information highlights the need for 3-D imaging, making 2-D imaging unnecessary. When there are questions about the soft tissue, the patient has to be referred to a specialist for computed tomography (CT) or magnetic resonance imaging (MRI).

### Image quality

In general, 3-D images are superior to 2-D images in terms of image quality, as the bone can be clearly visualized distortion-free in all planes [9, 12], there are no overlay effects, and the relationship to the surrounding structures is more evident [13, 14]. Additionally, the trabecular bone structure [15–20] and the need for augmentation can be evaluated more accurately in 3-D imaging [2, 9, 14]. Despite these advantages, in some studies, CBCT images appear to have errors that may exceed 1 mm in the measurements typically used for dental implants [21]. This accuracy seems to be similar with that of conventional CT [22–24]. One disadvantage of 3-D imaging compared with 2-D imaging is that the radiation exposure is usually higher with 3-D imaging [25].

### Radiation exposure

The effective dose of a standard panoramic radiograph is 2.7–24.5 μSv [26–30], versus 5 μSv for a single-tooth radiograph [30]. In contrast, the effective dose of a conventional CT scan ranges from 180-2100 μSv [31], and from 10–1000 μSv on CBCT [27, 30, 32]. The effective dose of CBCT for dentoalveolar imaging is 11–674 μSv [27, 30, 33, 34], and ranges from 11–96.2 μSv based on the scan protocols used for implant planning [35]. Such a wide range of effective doses puts an emphasis on patient- [36] and question-specific imaging protocol settings [28], such as low dose protocols [14, 35, 37], to follow the as low as diagnostically acceptable (ALADA) principle [8, 14, 37, 38].

### Visibility of anatomical structures in 3-D images

Many anatomical structures, such as the incisive nerve [35, 39, 40], the alveolar inferior nerve [41], and its anterior loop [40, 42], are more easily visualized on CBCT images [14] in contrast to 2-D images. Since measurement inaccuracies in CBCT scans can exceed 1 mm, a safety margin of 2 mm from vital structures should be utilized in implant planning [21, 37]. Although the peri-implant tissue can be visualized on CBCT images [43, 44], a single-tooth radiograph should be the first choice [14, 43, 45, 46].

### Indications for navigation for dental implantology

Dental implant placement guided by dynamic or static navigation is more accurate than the freehand technique [4, 14, 47, 48]. In digital implant planning, 3-D imaging of the implant site is integrated into a planning software, and the implant is virtually placed. This information can then be applied to the patient by static navigation via a drilling template or by dynamic navigation with live feedback of the position of the instrument in the patient’s mouth [49].

### Virtual planning

The 3-D images uploaded into the software enable the user to identify the structure and dimensions of the imaged bone. Virtual implant databases are part of the software and have information on the length, diameter, shape, and type of implants available. Therefore, the most suitable implant, site, and orientation can be selected. This information can be exported to plan the navigation.

### Dynamic navigation

For dynamic navigation, the patient has to wear a reference marker attached to a dental splint during image data acquisition. Intraoperatively, this marker serves as a reference point so that the orientation of the registered instrument can be displayed live on-screen [50]. This constant feedback enables the surgeon to place the implant with the precision that would be achieved with static navigation [51].

### Static navigation

A drilling template can be created from the data output from the planning software in a variety of ways, such as 3-D printing or computer-aided manufacturing. The drilling template can provide the position, depth, and angulation of the implant. Intraoperatively, the template can be supported by different tissues, although teeth- and gingiva-supported templates are more accurate when compared with bone-supported templates [52]. Transgingival implantation using a drilling guide reduces postoperative pain as well as the need for analgesics, when compared with open flap surgery [53]. Many patients, especially those with preexisting conditions such as bleeding disorders, may benefit from transgingival, template-guided implant placement; however, the availability of keratinized gingiva at the implant side must be sufficient [37].

### Accuracy of implant placement

When comparing freehand with static and dynamic navigation-guided implant placement, either type of navigation allows for more accurate placement than the freehand technique [4, 47, 48, 54–58]. A mean difference in angular deviation of − 5.54 ° and an apical deviation of 0.83 mm were calculated in a meta-analysis comparing navigation-guided vs. freehand placement [4]. Static and dynamic navigation techniques seem to be comparable in accuracy [51]. In randomized controlled trials, the deviation of the implant axis and the position of the implant tip for dynamically and statically navigated implants are as follows: 2.84 ± 1.71° and 1.28 ± 0.46 mm, and 3.06 ± 1.37° and 1.29 ± 0.5 mm, respectively [37, 48, 51].

### Implant survival

In randomized controlled trials, there was no evidence of any benefit for implants placed with navigational-guidance, particularly in terms of implant survival, peri-implant bone loss, or bleeding on probing after one and three years post-implantation [59]. After 5 years, clinically insignificant marginal bone loss was detected at implants placed with navigation [53]. Postoperative swelling and bleeding, however, were reduced [37, 53, 54, 59–61] due to the minimally invasive approach provided by navigation-guided dental implant placement [60].

## Discussion

The basis for safe implant placement is a good understanding of the patient’s anatomy. A 2-D image, in combination with a clinical examination, is sufficient in many cases. A panoramic X-ray with a reference body should be used when possible. While 3-D images are superior in terms of image quality [13], they also have a higher radiation dose and cost. Higher accuracy can be achieved with static or dynamic navigation guidance, when compared with freehand implant placement, although more preoperative planning is necessary [14]. The radiation exposure from 3-D imaging is generally higher than that of 2-D imaging; however, there is a wide range, based on the scanning protocol [26, 32, 62]. Comparing the radiation exposure of conventional CT scans with CBCT, conventional scans generally have higher doses, despite an overlapping range of some indications [34, 63]. For implantology, CBCT should be the first choice for 3-D imaging in most cases [14, 64]. Furthermore, radiation exposure is highly dependent on the equipment and settings utilized. A reduction in exposure can be achieved by shielding vulnerable tissues such as the thyroid gland [26], by reducing the field of view [65], the acquisition time(s), the tube voltage, or by increasing the voxel size [8, 32]. Many CBCT scanners have programmed these settings as low-dose protocols, which are typically sufficient to provide the information required for dental implantology [8, 14, 35, 37, 66].

In complex cases, 3-D images may be preferable [13, 67]. Examples of these instances are as follows: anatomical variations of the bone [68], insufficient visibility of vital structures on 2-D imaging, pathological changes visualized on 2-D imaging, pre-existing conditions, previous surgery in the maxillary sinus [14], for certain guided implantological methods [14], and for detecting possible complications after augmentation or implantation [13, 32, 69].

To achieve high accuracy with navigational-guidance, the workflow must be well established, since inaccuracies in each individual step can compound on each other [70]. For example, the positioning of the drilling template must be secured. To ensure a safe and correct implant placement, a 2-mm [21] safety margin adjacent to vital structures, such as the mandibular nerve, should be utilized during the planning process [13].

The use of navigation should be considered in the following situations: for special prosthetic techniques like immediate implant placement, as support for minimally invasive techniques, or after complex jaw reconstructions [71]. There were no studies found that indicated a reduction of adverse events, such as nerve damage, when using 3-D imaging or navigational-guidance. This is most likely due to the fact that adverse events occur very infrequently, and the number requiring treatment that is needed to show a significant difference is prohibitively high for a limited study population. Additionally, it is likely that more support tools were used at difficult implant sites, which may be another reason for the absence of comparative studies. Future studies evaluating these differences would be beneficial. No studies were present investigating whether a higher accuracy in the implants placed with navigational guidance leads to a higher long-term survival of the prosthetic. Further research in this regard would also be beneficial. The limitations of the present study were primarily due to the heterogeneity of the included studies. Furthermore, many of the described outcome parameters depend on the personal skills and experience of the surgeon.

## Conclusion

Although the availability of 3-D imaging is rapidly increasing, the temptation to utilize 3-D imaging in every implant placement has to be resisted. Despite the higher accuracy achieved in implants placed with the help of navigation, a difference in the survival of the implants has not been proven. In order to achieve the best possible outcome for the patient, the potential harm caused by radiation exposure also should be considered. Decisions regarding the imaging technique and the scanning protocols should be made on a case-by-case basis.

## Supplementary Information

**Additional file 1.** Evidence table.

## Data Availability

The datasets analyzed during the present study are available at Pubmed (https://pubmed.ncbi.nlm.nih.gov/).
