# Supplementary material for: Indications for 3-D diagnostics and navigation in dental implantology with the focus on radiation exposure: a systematic review
Source: Int J Implant Dent. 2021 May 27;7:52. doi: 10.1186/s40729-021-00328-9 (PMC8155130; doi:10.1186/s40729-021-00328-9)
Supplement: Supplementary file 1 — Additional file 1. Evidence table. [file 40729_2021_328_MOESM1_ESM.doc]

evidence table

| **reference** | **study type** | **participants** | | | **drop out quota** | **intervention** | **control** | **target parameter** | **main result** | **comments** | **degree of evidence-SIGN** |
| --- | --- | --- | --- | --- | --- | --- | --- | --- | --- | --- | --- |
|  |  | **over all** | | |  |  |  |  |  |  |  |
|  |  | **intervention group** | | **control group** |  |  |  |  |  |  |  |
| Conclusion: | | | | | | | | | | | |
| Kang, S. R.  Bok, S. C.  Choi, S. C.  Lee, S. S.  Heo, M. S.  Huh, K. H.  Kim, T. I.  Yi, W. J.  2016  10.5051/jpis.2016.46.2.116 | experimental study |  | |  |  | cbct, micro CT |  | Implant stability in relationship to trabecular microstructure and density | There was an increase in implant stability prediction by combining BV/TV and SMI in the stepwise forward regression analysis. Bone with high volume density and low surface density shows high implant stability. Well-connected thick bone with small marrow spaces also shows high implant stability. |  | 2++ |
| Conclusion: The combination of bone density and architectural parameters measured using CBCT can predict the implant stability more accurately than the density alone in clinical diagnoses. | | | | | | | | | | | |
| Poeschl, P. W.  Schmidt, N.  Guevara-Rojas, G.  Seemann, R.  Ewers, R.  Zipko, H. T.  Schicho, K.  2013  10.1007/s00784-012-0704-6 | experimental  study | 10 photopolymer-acrylate mandibular models with 4 metal reference markers | | 10 photopolymer-acrylate mandibular models with 4 metal reference markers | 0 | cbct | mcst | distance measurement | distance measurement between CBCT and MCST not statistically different. Overall mean difference: CBCT 0,46mm; MSCT 0,43mm |  | 2++ |
| conclusion:  Concerning CBCT significantly lower radiation expo- sure, reasonably short scanning times, compact design together with adequate accuracy are the main advantages. The data of our study prove that the application of CBCT for the indicated purpose yielded good results comparable to those of MSCT. | | | | | | | | | | | |
| Stratis, A.  Zhang, G.  Lopez-Rendon, X.  Politis, C.  Hermans, R.  Jacobs, R.  Bogaerts, R.  Shaheen, E.  Bosmans, H.  2017  10.1016/j.ejmp.2017.03.027 | experimental study | ICRP reference voxel phantom | |  |  | cbct | mcst | effective dose in mSv | For orthognathic protocols, dental CBCT scanners deliver lower doses compared to MDCT scanners. The estimated effective dose (ED) was 0.32 mSv for a normal resolution operation mode in Promax 3D Max, 0.27 mSv in VGi-evo and 1.18 mSv in the Somatom Definition Flash. |  | 2++ |
| conclusion: Two clinical exams which are carried out with both a CBCT or a MDCT scanner were compared in terms of radiation dose. Dental CBCT scanners deliver lower doses for orthognathic patients whereas for temporal bone procedures the doses were similar. | | | | | | | | | | | |
| Pauwels, R.  2015  10.1093/rpd/ncv057 | narrative review |  | |  |  | cbct | msct | effective dose  indication of the cbct in implant dentistry | The widespread use of cone-beam CT (CBCT) in dentistry has led to increasing concern regarding justification and optimization of CBCT exposures. When used as a substitute to multidetector CT (MDCT), CBCT can lead to significant dose reduction; however, low-dose protocols of current-generation MDCTs show that there is an overlap between CBCT and MDCT doses. |  | 2+ |
| conclusion: CBCT can often lead to improved diagnosis and treatment compared with 2D radiographs, a routine or excessive use of CBCT would lead to a substantial increase of the collective patient dose. | | | | | | | | | | | |
| Leung, C. K. K.  Pow, E. H. N.  Li, T. K. L.  Lo, E. C. M.  Chow, T. W.  2017  10.1111/jicd.12232 | experimental study | six pig ribs at measured at 25 locations. | |  |  | cbct, msct | digital caliper | distance measurement | No differences were found between measurements made by CBCT and HCT images using the default software (P > 0.05). However, both measurements were statistically-significantly lower than the control (P < 0.001), and the mean difference was 0.3 mm. |  | 2++ |
| conclusion: The accuracy of CBCT and HCT are similar, and both are reliable tools for implant planning | | | | | | | | | | | |
| Bohner, L. O. L.  Mukai, E.  Oderich, E.  Porporatti, A. L.  Pacheco-Pereira, C.  Tortamano, P.  De Luca Canto, G.  2017  10.1016/j.oooo.2017.06.119 | systematic review | 12 papers included | |  |  | cbct, ct | intraoral radiograph, panoramic images | assessment of periimplant bone defects | The sensitivity for CBCT was 59%, whereas the specificity was 67%. For IR, the sensitivity was 60% and the specificity was 59%. |  | 1- |
| conclusion: Both CBCT and IR showed a clinically acceptable performance for assessing peri-implant bone defects. | | | | | | | | | | | |
| Sheridan, R. A.  Chiang, Y. C.  Decker, A. M.  Sutthiboonyapan, P.  Chan, H. L.  Wang, H. L.  2018  10.1097/id.0000000000000684 | experimental study | 19 implants placed in 9 fresh frozen cadavers | | 19 implants removed in 9 fresh frozen cadavers |  | cbct |  | distantace measurement | The measurements of the cross- sectional images before and after implant removal are recorded in Table 1. The mean differences between the initial and postimplant removal CBCT scans for each of the 8 measurements are recorded in Table 2. No statistically significant differences (P . 0.05) were found. |  | 2+ |
| conclusion: The presence of dental implants did not impact the accuracy of CBCT measurements of bone thickness by the metallic artifact with this specific machine used for this study. | | | | | | | | | | | |
| Yepes, J. F.  Al-Sabbagh, M.  2015  10.1016/j.cden.2014.09.003 | narrative review |  | |  |  | cbct | panoramic images, periapical radiographs | the role of the cbct in detecting early implant failure | Traditional image modalities, such as periapical radiographs, bitewing radiographs, and panoramic radiographs, perform at least as well as CBCT when the goal is evaluation of implant placement |  | 2++ |
| conclusion: For patients with postoperative complications, diagnostic imaging, including CBCT, may be indicated as a supplement to the clinical examination. | | | | | | | | | | | |
| Dave, M.  Davies, J.  Wilson, R.  Palmer, R.  2013  10.1111/j.1600-0501.2012.02473.x | experimental study | Implants placed in fresh bovine rips | |  |  | cbct | long cone periapical radiographs (LCPAs) | subjective rating with a five-point scale | Digital LCPAs were better at diagnosing a peri-implant bone defect when the peri-implant space was 0.35 mm (P < 0.02). As the peri-implant space increased to 0.675 mm, there was no significant difference in diagnostic accuracy between the three imaging methods. |  | 2++ |
| conclusion: Within the limitations of this study, LCPAs are a reliable and valid method of detecting circumferential peri-implant bone defects and performed significantly better than CBCT. | | | | | | | | | | | |
| Mercado, F.  Mukaddam, K.  Filippi, A.  Bieri, O. P.  Lambrecht, T. J.  Kuhl, S.  2019/03/19  DOI:10.11607/jomi.7076 | experimental study | 5 upper jaws of pigs with 6 osteotomies each | | literature | 0 | -mri and intraoral scan.  -osteotomy |  | accuracy of the osteotomy | angular deviation of 3,23 mm; apical deviation 1,76mm, crestal deviation 1,76mm | no control group | 2+ |
| conclusion: The work flow described in this study represents a new and alternative way of performing guided implant surgery in edentulous cases. The accuracy calculated is comparable to the alternative work flows for guided implant surgery in edentulous cases. | | | | | | | | | | | |
| Fokas, G.  Vaughn, V. M.  Scarfe, W. C.  Bornstein, M. M.  2018  10.1111/clr.13142 | systematic review | 22 Studies included: 2 clinical 20 ex vivo | |  |  | cbct | physical measurements | linear measurements on cbct images | voxel size: 0,3-0,4 mm adequate for dental implant placement | artifacts not taken into account | 1+ |
| conclusion: CBCT can be considered as an appropriate diagnostic tool for 3D preoperative planning. Nevertheless, a 2-mm safety margin to adjacent anatomic structures should be considered when using CBCT. | | | | | | | | | | | |
| Pauwels, R.  Jacobs, R.  Singer, S. R.  Mupparapu, M.  2015  10.1259/dmfr.20140238 | systematic review | 34 studies included | |  |  | cbct | msct | gray value in Hounsfield Units | gv are to unreliable to detect bone density |  | 1+ |
| conclusion: GVs in CBCT should be generally avoided owing to its unreliability. | | | | | | | | | | | |
| Bornstein, M. M.  Scarfe, W. C.  Vaughn, V. M.  Jacobs, R.  2014  DOI: 10.11607/jomi.2014suppl.g1.4 | systematic review | 1.question:  (12 studies)  2.question:  (44 studies)  3. question:  (22 studies) | |  |  | cbct | orthopantomogram | 1. guideline for cbct and implants  2. contra-/ indication for cbct and dental implant  3. radiation dose risk for cbct and dental implant | 1. 12 studies exist with various qualities  2. various recommendations are made  3. dose risk is higher in cbct then opt |  | 1+ |
| conclusion:  1. Most published guidelines on implant dentistry do not offer evidence- based action statements developed from a rigorous systematic review approach.  2. Difficult to proof benefit of cross-sectional imaging over conventional two- dimensional with respect to damage of neurovascular structures arches resulting in dysesthesia in comparative prospective studies due to the high number to treat.  3. The effective doses for different CBCT devices exhibit a wide range. Significant dose reduction can be achieved by reducing the FOV. | | | | | | | | | | | |
| Jacobs, R.  Salmon, B.  Codari, M.  Hassan, B.  Bornstein, M. M.  2018  DOI: 10.1186/s12903-018-0523-5 | narrative review |  | |  |  | cbct |  | -cbct use  -implant oriented application  -presurgical imaging  -postsurgical imaging  -image quality  -dose evaluation  Implant planning | cbct is indicated in implant dentistry | selection criteria for the included publications not presented | 2++ |
| conclusion: Cbct scanning is increasing because of the popularity of computer-guided surgery.  CBCT imaging following insertion of dental implants should be restricted to specific post- operative complications. | | | | | | | | | | | |
| Rehani, M. M.  Gupta, R.  Bartling, S.  Sharp, G. C.  Pauwels, R.  Berris, T.  Boone, J. M.  2015  10.1177/0146645315575485 | guideline |  | |  |  | cbct |  | guideline for the use of the cbct with the focus of radiation exposure | cbct is indicated in implant dentistry |  | 1+ |
| conclusion: The smallest available volume size should always be chosen, as this could reduce patient dose considerably. The entire image should be examined and reported, not just the region of interest. | | | | | | | | | | | |
| Weiss, R., 2nd  Read-Fuller, A.  2019  DOI: 10.3390/dj7020052 | narrative review |  | |  |  | cbct |  | -anatomy  -bone quality and volume  -implant selection  -augmentation and site preparation procedures  -application of surgical guides and splints  -post-operative evaluation | - anatomical structures a better visualized  - there is no universally accepted bone quality grading system  - bone volume is assessed correctly  - implant selection corresponds more often with the plan when cbct is used  - in zygomatic implants cbct is necessary for planning the placement  - septation of the sinus can only reliably found in cbct  - implant guides are more accurate when cbct is used compared to free hand  - post operatively intraoral radiography should remain the main diagnostic imaging modality in monitoring implants post-operatively | Literature selection criteria is limited | 1+ |
| conclusion: Thera are only a few categories in which cbct significantly altered a predetermined plan: implant size selection and the necessity for bone augmentation at the time of implant placement. Limited data has quantified the ability of cbct to affect clinical outcome or complication rates. | | | | | | | | | | | |
| David Harris  Keith Horner  Kerstin Gro ̈ndahl Reinhilde Jacobs Ebba Helmrot  Goran I. Benic Michael M. Bornstein Andrew Dawood Marc Quirynen  2012  DOI:  10.1111/j.1600-0501.2012.02441.x | systematic review: guideline |  | |  |  |  |  | radiological and clinical considerations | radiological and clinical recommendations | no literature selection criteria | 1+ |
| conclusion: If the clinical assessment of implant sites indicates that there is sufficient bone width and the conventional radiographic examination reveals the relevant anatomical boundaries and adequate bone height and space, no additional imaging is required for implant placement. | | | | | | | | | | | |
| Ferreira Barbosa, D. A.  Barros, I. D.  Teixeira, R. C.  Menezes Pimenta, A. V.  Kurita, L. M.  Barros Silva, P. G.  Gurgel Costa, F. W.  2019  doi:10.11607/jomi.6730 | systematic review | 25 studies included | |  |  | cbct |  | mic (mandibular incisive canal) | -The overall mean prevalence of MIC was 89.6% ± 15.08%  - The studies using a voxel size lower than 0.3 mm showed the highest mean prevalence (93.88%) in comparison with voxel size described as ≥ 0.3 mm (89.33%). |  | 1+ |
| conclusion: There is a high worldwide prevalence of mic. | | | | | | | | | | | |
| Yeung, A. W. K.  Jacobs, R.  Bornstein, M. M.  2019  DOI: 10.1007/s00784-019-02907-y | narrative review | 27 studies included | |  |  | cbct low dose |  | effect of low dose protocols on implant planning | A retrospective clinical study and concluded that reducing the mA and exposure time and the use of a half-scan can be used for implant planning without any loss in diagnostic value or efficacy for treatment planning purposes. These findings are largely confirmed by multiple studies using skulls or cadavers (Table 4). With regard to the detection of peri-implant bone loss, dose reduction to diagnose fenestration- and dehiscence-type defects can be achieved by using larger voxel sizes, and for fenestrations a half-scan is recommended |  | 1+ |
| conclusion: A low-dose protocol should be considered in various disciplines in dental medicine, specifically in implant dentistry (planning implant insertion, evaluating peri-implant bone loss).  Dose reduction is usually achieved by mAs reduction, use of partial rotations, reduced number of projections, and larger voxel size, but seldom by kV reduction. | | | | | | | | | | | |
| A Dawood*^,1,2^, J Brown^3^, V Sauret-Jackson1 and S Purkayastha^1^  2012  doi: 10.1259/dmfr/16421849 | retrospective practice-based study |  | |  |  | cbct with high radiation exposure protocols | cbct with low radiation exposure | subjective image quality rated by 6 expierenced surgeons | Lower-dose protocols only marginally affected the preference of the reviewers of the resulting images. |  | 2++ |
| Conclusion: There is potential to reduce patient dose very significantly in CBCT examinations for implant site evaluation. | | | | | | | | | | | |
| Beatrice Feragalli1 · Osvaldo Rampado2 · Cecilia Abate3 · Monica Macrì1 · Felice Festa1 · Francesco Stromei4 · Sergio Caputi1 · Giuseppe Guglielmi^3^,**5**  2017  10.1007/s11547-017-0758-2 | experimental study |  | |  |  | cbct with high radiation exposure protocols | cbct with low radiation exposure  protocols | Image quality | CBCT low-dose protocol with large FOV, normal resolution quality images, 80 kVp, 5 mA and acquisition time of 15 s resulted in a value of effective dose of 35 microSievert (μSv). |  | 2++ |
| Conclusion: CBCT performed with low-dose protocol has a very low radiation exposure and good quality of images and, therefore, could be proposed for dental, orthodontic and maxillofacial studies in cases where a complete evaluation of the maxillofacial region is essential in order to set the proper treatment. | | | | | | | | | | | |
| Tyndall, D. A.  Price, J. B.  Tetradis, S.  Ganz, S. D.  Hildebolt, C.  Scarfe, W. C.  2012  DOI: 10.1016/j.oooo.2012.03.005 | guideline: research based consensus |  | |  |  | cbct, intraoral radiography, cephalometric radiography, panoramic radiography, ct, conventional tomography |  | dental implant placement | Factors reducing radiation exposure: device-based—scan angle, additional copper filter, FOV diameter, FOV height, kV, mA, exposure time, mAs, voxel, and resolution; (2) non-device- based—patient size, region of interest, and use of thyroid shield |  | 1++ |
| conclusion: Initial imaging assessment is achieved with panoramic radiography and may be supplemented with periapical radiography.  For the preoperative diagnostic phase, the AAOMR reaffirms that cross-sectional imaging be used for implant site assessment. | | | | | | | | | | | |
| Tavelli, L.  Borgonovo, A. E.  Re, D.  Maiorana, C.  2017  DOI:  10.23736/s0026-4970.17.04027-4 | narrative review | 54 studies included | |  |  |  |  | establishment of a maxillary sinus classification prior to surgery | cbct is indicated for several additional diagnostic purposes  -eleven parameters, that clinicians must check every time through cbct or ct  - grading system was developed | little search criteria presented | 1+ |
| conclusion: It encourages the surgeon to have a careful preoperative evaluation through cbct, which is believed to be highly essential. | | | | | | | | | | | |
| Rios, H. F.  Borgnakke, W. S.  Benavides, E.  2017  DOI: 10.1902/jop.2017.160548 | narrative review | 161 papers included | |  |  | cbct |  | cbct use in implant dentistry | Literature supports the accuracy of CBCT in the following aspects:  1) linear measurements of the available ridge height, width, and relative bone quality; 2) assessment of 3D surface alveolar ridge topography; 3) characterization of vital anatomic structures relevant to the implant site; and 4) recognition of incidental pathology | little literature research criteria presented | 1+ |
| conclusion: Cbct continues to be considered an advanced point-of-care imaging modality and should be used selectively as an adjunct to two-dimensional dental radiography. | | | | | | | | | | | |
| Wismeijer, D.  Joda, T.  Flugge, T.  Fokas, G.  Tahmaseb, A.  Bechelli, D.  Bohner, L.  Bornstein, M.  Burgoyne, A.  Caram, S.  Carmichael, R.  Chen, C. Y.  Coucke, W.  Derksen, W.  Donos, N.  El Kholy, K.  Evans, C.  Fehmer, V.  Fickl, S.  Fragola, G.  Gimenez Gonzales, B.  Gholami, H.  Hashim, D.  Hui, Y.  Kokat, A.  Vazouras, K.  Kuhl, S.  Lanis, A.  Leesungbok, R.  van der Meer, J.  Liu, Z.  Sato, T.  De Souza, A.  Scarfe, W. C.  Tosta, M.  van Zyl, P.  Vach, K.  Vaughn, V.  Vucetic, M.  Wang, P.  Wen, B.  Wu, V.  2018  10.1111/clr.13309 | systematic reviews and consensus report | 232 articles included | |  |  | cbct |  | accuracy of placed implants | Static computer-aided surgery (s-CAIS), in terms of pain & discomfort, eco- nomics and intraoperative complications, is beneficial compared with conventional implant surgery.  When using s-CAIS in partially edentulous cases, a higher level of accuracy can be achieved when compared to fully edentulous cases. |  | 1+ |
| conclusion: Because the precision intraoral scans and of measurements on CBCT scans and is not high enough to allow for the required accuracy, s-CAIS should be considered as an additional tool for comprehensive diagnosis, treatment planning, and surgical procedures | | | | | | | | | | | |
| Benavides, E.  Et al.  2012  DOI: 10.1097/ID.0b013e31824885b5 | narrative review : consensus report |  | |  |  | cbct |  | Cbct in implant dentistry | Potential indications for CBCT:  Computer-aided implant planning  • Implant placement in a highly esthetic zone or where concavities, ridge inclination, inadequate bone volume or quality, undeterminable proximity to vital structures, and insufficient inter-radicular spacing is suspected  • Pre- and post advanced bone grafting evaluation  • History or suspected trauma to the jaws, foreign bodies, maxillofacial lesions, and/or developmental defects  • Evaluation of post implant complications | little literature research criteria presented | 1+ |
| conclusion: Cbct examinations, as all other radiographic examinations, must be justified on an individualized needs basis. | | | | | | | | | | | |
| Pires, C. A.  Bissada, N. F.  Becker, J. J.  Kanawati, A.  Landers, M. A.  2012  DOI: 10.1111/j.1708-8208.2009.00228.x | retrospective study |  | |  |  | cbct | panoramic radiography | imaging of the incisive nerve canal | Eighty-three percent of the CBCT scans showed the presence of the incisive canal, as did 11% of the panoramic radiographs. | little literature research criteria presented | 2++ |
| conclusion: The presence, location, and dimensions of the mandibular incisive canal are better determined by CBCT imaging than by panoramic radiography. | | | | | | | | | | | |
| Haas, L. F.  Dutra, K.  Porporatti, A. L.  Mezzomo, L. A.  De Luca Canto, G.  Flores-Mir, C.  Correa, M.  2016  10.1259/dmfr.20150310 | systematic review and metanalysis | 15 studies included | |  |  | cbct | panoramic  radiography | anatomical variations of mandibular canal | The overall prevalence of anatomical variations for in situ studies was 6.46%, and through assessment of panoramic radiography and CT or CBCT the overall prevalence shown was 4.20% and 16.25%, respectively. |  | 1+ |
| Conclusion: There are two types of variations of the mandibular canal: the retromolar canal and bifid mandibular canal. The frequency variations through assessing in situ, panoramic radiography and CT or CBCT were 6.46%, 4.20% and 16.25%, respectively. | | | | | | | | | | | |
| de Brito, A. C.  Nejaim, Y.  de Freitas, D. Q.  de Oliveira Santos, C.  2016  DOI: 10.5624/isd.2016.46.3.159 | retrospective analysis | 90 patients | |  |  | cbct | panoramic radiographs | extension of the anterior loop and mandibular incisive canal | In cbct, the anterior loop and the incisive canal were observed in 7.7% and 24.4% of the hemimandibles, respectively. In PAN, the anterior loop and the incisive canal were detected in 15% and 5.5% of cases, respectively. |  | 2+ |
| conclusion:  This review has found no evidence to support any specific imaging modality when planning dental implant placement in any region of the mouth. | | | | | | | | | | | |
| Pertl, L.  Gashi-Cenkoglu, B.  Reichmann, J.  Jakse, N.  Pertl, C.  2013 | experimental study |  | |  |  | cbct | ct and panoramic radiography | linear accuracy | If steel balls were used during OPG, the median distortion was lowered to 0.2 mm, but the width of -1.6 to 3 mm was still quite extensive.  CT images showed a mean distortion of -0.2 mm and a width of -1.5 to 1.3 mm. The mean distortion of the CBCT images was similar to the one found in CT, namely -0.3 mm with a range from -1.5 to 0.8 mm |  | 2+ |
| conclusion: The results show that OPG using steel balls as a calibration reference seems reliable in a standard situation. | | | | | | | | | | | |
| Shelley, A. M.  Glenny, A. M.  Goodwin, M.  Brunton, P.  Horner, K.  2014  doi:10.1259/dmfr.20130321 | systematic review | 5 papers included | |  |  | cbct; ct | panoramic radiography | do preoperative 3d images change the outcome | Little can be determined from a synthesis of the studies because of their small number, clinical diversity and high risks of bias. Notwithstanding, it may be tentatively inferred that cross-sectional imaging has a therapeutic impact in the more challenging cases |  | 2+ |
| conclusion:  This review has found no evidence to support any specific imaging modality when planning dental implant placement in any region of the mouth. | | | | | | | | | | | |
| Jensen, C.  Raghoebar, G. M.  Meijer, H. J.  Schepers, R.  Cune, M. S.  2016  DOI:10.1111/cid.12359 | retrospective analysis | 44 patients included | |  |  | cbct | panoramic radiographs and stone cast | Implant placement in the resorbed posterior mandible | -Attempting implant placement solely on the basis of a panoramic radiograph could have resulted in damage to vital tissues in 4% of cases (false-positive rate). |  | 2+ |
| conclusion: The degree of consistency between the two diagnostic procedures, panoramic radiograph, and cbct is observer dependent. Justification for ordering a cbct will depend on the willingness of both patient and clinician to accept the number of misclassifications, which amount to approximately 10% to 13% of the observations, dependent on the area. | | | | | | | | | | | |
| Sahota, J.  Bhatia, A.  Gupta, M.  Singh, V.  Soni, J.  Soni, R.  2017 | retrospective analysis | 100 panoramic radiographs | | 100 cbcts |  | cbct | panoramic radiography | distance between the roots of the lateral teeth and the inferior alveolar nerve canal | There was no statistically significant difference in the mean distance from the root of the second premolar and the mesial and distal roots of the first molar to the IANC between PR and CBCT images. The difference in the mean distance from the mesial and distal roots of the second and the third molars to the IANC measured in PR and CBCT images was statistically significant. |  | 2+ |
| conclusion: PR may be uninformative or misleading when measuring the distance from the mesial and distal roots of the second and the third molars to the IANC. | | | | | | | | | | | |
| Tang, Z.  Liu, X.  Chen, K.  2017  DOI: 10.1186/s13005-017-0135-3 | retrospective analysis | 86 cbct/pr | | 86cbct/pr |  | cbct | panoramic radiography | distance measurement at different locations | The present study showed different magnification rates of OPG compared with CBCT in measuring different maxillofacial loci. There were highly related correlation coefficients (R) between the paired samples obtained from OPG and CBCT. |  | 2- |
| conclusion: The magnification rates of opg at these teeth are different. The distances measured by opg were highly correlated with that measured by cbct | | | | | | | | | | | |
| Shahidi, S.  Zamiri, B.  Abolvardi, M.  Akhlaghian, M.  Paknahad, M.  2018 | retrospective analysis | 132 cbct | | 508 panoramic radiography |  | cbct | panoramic radiography | distance measurement in the posterior segment of the mandible | The mean error of DPR in detecting the available bone height for edentulous ridges in posterior of mandible was 0.21mm±0.42mm, which did not make a serious confusion in pre-surgical planning of dental implant therapy, especially in routine cases. |  | 2+ |
| conclusion: Panoramic radiography can be used safely in the pre-surgical phase of dental implantation in the posterior mandible especially in routine and simple cases when cbct is not available. | | | | | | | | | | | |
| Elshenawy, H.  Aly, W.  Salah, N.  Nasry, S.  Anter, E.  Ekram, K.  2019  DOI: 10.3889/oamjms.2019.232 | experimental study | 13 models and cbct | | 13 models with real linear measurement |  | cbct |  | effect of changing FOV and dimensional accuracy | There is an increase in measurement error with increase FOV |  | 2++ |
| conclusion: cbct scans made with smaller FOVs and voxel sizes are associated with higher linear measurements accuracy than those made with larger FOVs and voxel sizes. For the same voxel size, smaller FOVs are associated with higher cbct linear measurements accuracy than those made with larger FOVs | | | | | | | | | | | |
| Grunheid, T.  Kolbeck Schieck, J. R.  Pliska, B. T.  Ahmad, M.  Larson, B. E.  2012  DOI: 10.1016/j.ajodo.2011.10.024 | experimental study | head and neck phantom | |  |  | cbct | panoramic  radiography | effective radiation doses | The effective doses at various voxel sizes and field of view settings ranged from 64.7 to 69.2 mSv for standard resolution CBCT scans (scan time 8.9 s) and 127.3 to 131.3 mSv for high resolution full field of view scans (scan time 17.8 s), and measured 134.2 mSv for a high- resolution landscape scan with a voxel size as would be used for SureSmile (OraMetrix, Richardson, Tex) therapy (scan time 26.9 s). The effective doses for digital panoramic and lateral cephalometric radiographs measured 21.5 and 4.5 mSv, respectively |  | 2++ |
| conclusion: cbct, although providing additional diagnostic and therapeutic benefits, also exposes patients to higher levels of radiation than conventional digital radiography. | | | | | | | | | | | |
| Al-Okshi, A.  Nilsson, M.  Petersson, A.  Wiese, M.  Lindh, C.  2013  10.1259/dmfr.20120343 | experimental study | head and neck phantom | |  |  | cbct | panoramic  radiography | effective radiation doses | The lowest effective dose of a cbct unit was observed for ProMax 3D, FOV 4 3 5 cm (10 mSv), the highest for NewTom VGi, FOV 8 3 8 cm—high resolution (129 mSv). The range of effective doses for digital panoramic machines measured was 8–14 mSv. |  | 2++ |
| Conclusion: The use of small FOV and standard resolution reduces the dose when compared with larger FOVs of the same ROI or higher resolution. | | | | | | | | | | | |
| Shin, H. S.  Nam, K. C.  Park, H.  Choi, H. U.  Kim, H. Y.  Park, C. S.  2014  10.1259/dmfr.20130439 | experimental study | head and neck phantom | |  |  | cbct | panoramic  radiography | effective radiation doses  and dose area product | the maximum effective doses from the Alphard 3030 and Rayscan Symphony were 67 and 21 times greater than that from panoramic radiography, respectively |  | 2++ |
| conclusion: adequate mode selection and control of exposure as well as further research are necessary to minimize the effective dose to patients, especially for radiosensitive children. | | | | | | | | | | | |
| Signorelli, L.  Patcas, R.  Peltomaki, T.  Schatzle, M.  2016  doi: 10.1007/s00056-015-0002-4 | experimental study | head and neck phantom | |  |  | cbct | conventional set of orthodontic radiographs (COR),  conventional lateral (LC) and poster anterior (PA) cephalograms and digital panoramic radiograph (OPG) | effective radiation doses | The following radiation levels were measured: 131.7, 91, and 77 lSv in the portrait, normal landscape, and fast landscape modes, respectively. The overall effective dose for a COR was 35.81 lSv (PA: 8.90 lSv; OPG: 21.87 lSv; LC: 5.03 lSv). |  | 2++ |
| conclusion: Cbct should not be recommended for use in all orthodontic patients as a substitute for a conventional set of radiographs. In cbct, reducing the height of the field of view and shielding the thyroid are advisable methods and must be implemented to lower the exposure dose | | | | | | | | | | | |
| Qiang, W.  Qiang, F.  Lin, L.  2019  DOI: 10.1093/rpd/ncy159 | experimental study | head and neck phantom | |  |  | Cbct | panoramic  radiography | effective radiation doses | For cbct, the organs are brain (0.636mGy), salivary glands (7.775mGy), thyroid (8.727mGy) and crystalline lens (4.022mGy). For panoramic machine, the organ are salivary glands (0.622 mGy) and thyroid (0.256 mGy) and for intraoral round cone device the organ is salivary glands (0.803 mGy). |  | 2++ |
| conclusion: CBCT scan can cause much higher effective dose than the other two. Brain, salivary glands, thyroid and the lens of the eye are tissues receiving relatively higher absorbed doses. | | | | | | | | | | | |
| Ibrahim, N.  Parsa, A.  Hassan, B.  van der Stelt, P.  Wismeijer, D.  2013  DOI: 10.1259/dmfr.20120075 | narrative review |  | |  |  | cbct | micro CT; mri; dental radiographs; high-resolution peripheral CT | bone microstructure | The use of CBCT could prove appealing. As the need to evaluate the implant insertion sites prior to surgical placement has dramatically increased, CBCT should be validated as a non-invasive procedure for assessing bone microstructure. |  | 2++ |
| conclusion: The current literature regarding diagnostic imaging assessment of trabecular microstructure prior to oral implant placement and suggests cone beam CT as a method of choice for evaluating trabecular bone microstructure. | | | | | | | | | | | |
| Jane-Salas, E.  Rosello, LLabres X.  Jane-Palli, E.  Mishra, S.  Ayuso-Montero, R.  Lopez-Lopez, J.  2018 10.1007/s10266-018-0343-8 | a randomized controlled pilot trial | 15 patients | | 15 patients |  | mucoperiostal flap elevation | flapless surgical technic | oral hygiene, pain, analgesics consumption, interincisal mouth opening,  implant success rate | Oral hygiene index, maximum interincisal opening, pain and analgesic consumption values had a signicant difference between groups favoring the apless technique at 24 h and 7 days but at the 15 days’ follow-up the di erences were only signi cant for oral hygiene and pain (P < 0.05) |  | 1+ |
| conclusion: Participants operated for implant placement with apless surgical technique go through less postoperative discomfort. Both techniques show high success rates, but to perform a apless technique patients must be properly selected. | | | | | | | | | | | |
| Vercruyssen, M.  Cox, C.  Coucke, W.  Naert, I.  Jacobs, R.  Quirynen, M.  2014  DOI: 10.1111/jcpe.12231 | randomized controlled trial | 72 Patients (jaws) requiring four to six implants | |  |  | Guides surgery mucosal or bone supported; | Mental navigation; pilot drill system | accuracy of implant placement | A significant lower mean deviation at the entry point (1.4 mm, range: 0.3-3.7), at the apex (1.6 mm, range: 0.2-3.7) and angular deviation (3.0°, range: 0.2-16°) was observed for the guiding systems when compared to mental navigation (2.7 mm, range: 0.3- 8.3; 2.9 mm, range: 0.5-7.4 and 9.9°, range: 1.5-27.8) and to the surgical template group (3.0 mm, range: 0.6- 6.6; 3.4 mm, range: 0.3-7.5 and 8.4°,range: 0.6-21.3°). Differences between bone and mucosa support or type of guidance were negligible. |  | 1+ |
| conclusion: Based on these findings one can conclude that guided surgery has an added value, but at each step awareness for possible errors in deviation is crucial for treatment success. | | | | | | | | | | | |
| Block, M. S.  Emery, R. W.  2016  DOI: 10.1016/j.joms.2015.09.022 | narrative review |  |  | |  | static and dynamic navigation | free hand navigation | accuracy and practical aspect of implant placement | A CT-generated static guide is recommended for edentulous cases. Dynamic navigation requires registration of the jaw to the navigation system, which currently cannot use intrabony fiducial markers.  However, dynamic navigation is indicated for any of the following:  1. Placement of implants in patients with a limited mouth opening.  2. Placement of the implant on the same day of the CBCT scan.  3. Placement of implants in difficult-to-access locations such as the second molar.  4. Placement of implants when direct visualization will be difficult.  5. Placement of implants in tight interdental spaces when static guides cannot be used owing to tube size.  6. Placement of implants adjacent to natural teeth in situations in which static guide tubes will interfere with ideal implant placement. |  | 2++ |
| conclusion:  either method will be advantageous compared with the freehand method. The choice of static or dynamic navigation will depend on the clinician’s preference and experience. | | | | | | | | | | | |
| D'Haese, J.  Ackhurst, J.  Wismeijer, D.  De Bruyn, H.  Tahmaseb, A.  2017  DOI: 10.1111/prd.12175 | narrative review |  |  | |  | dynamic and static navigation;  flapless | freehand navigation;  flap surgery | accuracy; implant survival | High levels of inaccuracies are reported where these techniques were applied. This imprecision seems most significant when bone-supported guides are used. The accuracy of these systems depends on all the cumulative and interactive errors involved, from data-set acquisition to the surgical procedure. | little literature research criteria presented | 2++ |
| conclusion: based on the available literature it can be concluded that no decisive evidence yet exists which suggests that computer-assisted surgery is superior to conventional procedures in terms of safety, treatment out- comes, morbidity or efficiency. | | | | | | | | | | | |
| Tannyhill, R. J., 3rd  Troulis, M. J.  2019  DOI: 10.1016/j.coms.2018.12.002 | narrative review |  |  | |  |  |  | rehabilitation of the cleft | Plain film, CT, or cone beam computed tomography images allow for evaluation of the outcome of previous grafting of the cleft site. | little literature research criteria presented | 2++ |
| conclusion: The complexity of the challenges in the cleft patient are minimized with a meticulous clinical and radiographic workup. Treatment planning is much more accurate with the use of advanced imaging, | | | | | | | | | | | |
| Block, M. S.  Emery, R. W.  Cullum, D. R.  Sheikh, A.  2017  DOI: 10.1016/j.joms.2017.02.026 | prospective cohort study |  |  | |  | fully guided (FG) and partially guided (PG) dynamic navigation | free hand navigation | accuracy of implant placement | Dynamic navigation will improve accuracy and precision of implant placement. Angulation deviation was the most important measurement improved using dynamic navigation. |  | 2++ |
| conclusion: Accuracy and precision for implant placement were achieved using dynamic navigation. The use of this type of method results in smaller deviations from the planned placement compared with FH approaches. | | | | | | | | | | | |
| Choi, W.  Nguyen, B. C.  Doan, A.  Girod, S.  Gaudilliere, B.  Gaudilliere, D.  2017  DOI: 10.1097/id.0000000000000620 | retrospective study | 450 implants placed |  | |  | free hand placed implants |  | factors influencing the accuracy of implant placement | The most important predictors of angulation and position accuracy were the number of adjacent implants placed and the tooth-borne status of the site. Immediate placement also significantly increased position accuracy, whereas cases with narrow sites were significantly more accurate in angulation. Accuracy also improved with the practitioner’s experience. |  | 2++ |
| conclusion: tooth-borne, single-implant cases performed later in the practitioner’s experience are most appropriate for freehand placement, whereas guided surgery should be considered to improve accuracy for multiple-implant cases in edentulous or partially edentulous sites | | | | | | | | | | | |
| Raico Gallardo, Y. N.  da Silva-Olivio, I. R. T.  Mukai, E.  Morimoto, S.  Sesma, N.  Cordaro, L.  2017  10.1111/clr.12841 | systematic review and meta-analysis |  |  | |  | computer aided implant surgery when using different supporting tissues |  | accuracy of the placed implant | The mucosa-supported guides indicated a statistically significant greater reduction in angle deviation (P = 0.02), deviation at the entry point (P = 0.002), and deviation at the apex (P = 0.04) when compared to the bone-supported guides. Between the mucosa- and tooth-supported guides, there were no statistically significant differences for any of the outcome measures. |  | 1+ |
| Conclusion: An important finding of this meta-analysis was that the bone-supported guides provided lower accuracy than did the tooth- and mucosa-supported guides. | | | | | | | | | | | |
| Alevizakos, V.  Mitov, G.  Stoetzer, M.  von See, C.  2019  DOI: 10.1016/j.oooo.2019.01.009 | retrospective study | 20 guided | 21 freehand placed implants | |  | guided implant placement | freehand implant placement | accuracy of the dental implant placement | The dental navigation system used in this retrospective study had significantly higher accuracy and precision than did freehand implantation for the angulation, basal and apical implant position, compared with the preoperative planning. |  | 2++ |
| conclusion: For inexperienced clinicians, the use of surgical templates during implantation procedures can be highly recommended, in order to increase the predictability and safety of the treatment. | | | | | | | | | | | |
| Kaewsiri, D.  Panmekiate, S.  Subbalekha, K.  Mattheos, N.  Pimkhaokham, A.  2019  DOI:  10.1111/clr.13435 | randomized controlled trial | 30 static | 30 dynamic | |  | static computer-assisted implant surgery | dynamic computer assisted implant surgery | accuracy of the dental implant placement | The deviation of implants toward the mesial direction in dynamic CAIS group was significantly higher than that of the static CAIS (p = 0.032). |  | 1++ |
| conclusion: Implant placement accuracy in single tooth space using dynamic CAIS appear to be the same to that of static CAIS. | | | | | | | | | | | |
| Smitkarn, Palita  Subbalekha, Keskanya  Mattheos, Nikos  Pimkhaokham, Atiphan  2019  DOI:10.1111/jcpe.13160. | randomized controlled trail | 30 static | 30 freehand | |  | static computer assistet implant surgery | freehand placement | accuracy of the dental implant placement in a single edentulous space | The median(IQR) deviations in angles, shoulders and apexes were 2.8(2.6)°, 0.9(0.8) mm and 1.2(0.9) mm, respectively, in the static CAIS group, and 7.0(7.0)°, 1.3(0.7) mm and 2.2(1.2) mm, respectively, in the freehand group. |  | 1++ |
| conclusion: Static CAIS provided more accuracy in implant positions than freehand placement in a single edentulous space. | | | | | | | | | | | |
| Stefanelli, L. V.  DeGroot, B. S.  Lipton, D. I.  Mandelaris, G. A.  2019  10.11607/jomi.6966 | retrospective observational study | 131 implants placed |  | |  | dynamic navigation |  | accuracy of implant placement | For all implants, the mean deviations were 0.79 (range 0.02-2.78) mm at entry (lateral), 1.10 (0.32-3.05) mm at apex (3D) and the mean angle was 2.59° (0.18°-8.99°). |  | 2+ |
| conlsuion: Computer aided implantology (CAI), when practiced in a flapless approach, has been demonstrated to provide many clear advantages over the free-hand unguided approach. | | | | | | | | | | | |
| Pozzi, Alessandro  Tallarico, Marco  Marchetti, Massimiliano  Scarfo, Bruno  Esposito, Marco  2014 | randomized controlled trial | 25 Patient with two implants each | 26 patients with two implants each | | 1 implant did not osseointegrate | freehand placement | computer-guided placement | prosthesis and implant failures, complications, peri-implant bone level changes, number of treatment sessions, duration of treatment, post-surgical pain and swelling, consumption of pain killers, treatment time, time required to solve complications, additional treatment cost, patient satisfaction | statistically significant differences between the two groups for any of the tested outcomes with the exception of more postoperative surgical pain (P = 0.002) and swelling (P = 0.024) at conventionally treated patients |  | 1++ |
| conclusion: When treatment planning was made on 3D CBTC scan using a dedicated software, no statistically significant differences were observed between computer-guided and a free-hand rehabilitations, with the exception of more postoperative pain and swelling at sites treated freehand because more frequently flaps were elevated. | | | | | | | | | | | |
| Bernard, L.  Vercruyssen, M.  Duyck, J.  Jacobs, R.  Teughels, W.  Quirynen, M.  2019  10.1016/j.prosdent.2018.09.004 | randomized controlled trail | 72 jaws were randomly (of 60 Patients) assigned to one of 6 groups  4 guided groups | | 2 non guided groups | 3 patients dropped out. 69 jaws were included in the 3 year follow up. | freehand placement | guided placement | marginal bone loss  Bop  pocket depth  plaque | No significant differences in bone loss were observed (P>.05).  No statistical difference between groups or follow- up period was found (P>.1) | The present study had insufficient power to measure survival differences between the groups. A longer follow- up time and larger groups are suggested for future research. | 1++ |
| Concusion: Within the limitation of this study, no statistically significant differences could be found between the guided group and the control group at the 3-year follow-up. | | | | | | | | | | | |
| Tallarico, Marco  Esposito, Marco  Xhanari, Erta  Caneva, Marco  Meloni, Silvio Mario  2018 | randomized controlled trial | 10 patients (32 implants) | | 10 patients (32 implants) | 1 patient from each group dropped out | freehand placement | computer-guided placement | Number of sessions from patient's recruitment to delivery of the definitive prosthesis, number of days from the initial CBCT scan to implant placement, consumption of painkillers, averaged surgical, prosthetic, and complication times,  Implant failure, pain, marginal bone loss, | There were no  Five years after loading, the mean marginal bone loss was 0.87 mm +/- 0.40 (95% CI: 0.54 to 1.06 mm) in the computer-guided group and 1.29 mm +/- 0.31 (95% CI: 1.09 to 1.51 mm) in the freehand group. The difference was statistically significant (difference 0.42 mm +/- 0.54; 95% CI: 0.05 to 0.75; P = 0.024). Patient self-reported post-surgical pain (P = 0.037) and swelling (P = 0.007) were found to be statistically significant higher in patients in the freehand group. |  | 1++ |
| conclusion: Both approaches achieved successful results over the 5-year follow-up period. Statistically higher post-operative pain and swelling were experienced at sites treated freehand with flap elevation. Less marginal bone loss (0.4 mm) was observed in the computer-guided group, at 5 years follow-up. | | | | | | | | | | | |
| Chen, S.  Ou, Q.  Lin, X.  Wang, Y.  2019  DOI: 10.1097/id.0000000000000915 | a systematic review and meta-analysis | 6 studies were included | |  |  | computer-aided surgical template | the free-hand method | accuracy of implants;  survival of implants | Comparison of the survival rate of implant surgery with or without an implant template revealed no significant result (OR = 1.71, 95% confidence interval [CI] 0.65-4.51). Significant differences in accuracy were observed in angular (mean difference = -5.45 degrees, 95% CI -0.66 to -4.24 degrees) and apical deviation (mean difference = -0.83 mm, 95% CI -1.12 to -0.54). |  | 1+ |
| Conclusion: With the technology of computer-aided surgical template, implant placement can be more accurate than free-hand operation. No significant difference is observed in the survival rate between template and free-hand. | | | | | | | | | | | |
| Suomalainen, A.  Pakbaznejad Esmaeili, E.  Robinson, S.  2015  10.1007/s13244-014-0379-4 | narrative review |  | |  |  | cbct | orthopanomogram  Single- tooth, radiograph | Indication of CBCT | • cbct imaging allows accurate 3D imaging of hard tissues.  • cbct offers lower costs and a smaller size and radiation  dose compared with MSCT.  • The disadvantages of cbct imaging are poor soft tissue  contrast and artefacts.  • The Sedentexct project has developed evidence-based guide-  lines on the use of cbct in dentistry. |  | 2++ |
| Conclusion: Panoramic radiography and intraoral radiography are still the basic imaging methods in dentomaxillofacial radiology and CBCT should be used in more demanding cases. | | | | | | | | | | | |
| Nicolielo LFP, Van Dessel J, van Lenthe GH, Lambrichts I, Jacobs R. The British journal of radiology. 2018;91(1092):20180437. | experimental study | Images of 25 cadaver mandibles. | | Images of 25 cadaver mandibles |  | cbct images without automatic classification | cbct images with automatic classification | automated classification method of trabecular bone pattern at implant site based on three-dimensional bone morphometric parameters | The overall correct classification was 83% for quantity-, 86% for structure-related parameters and 84% for the parameters combined. Cross-validation showed a 79% model prediction accuracy. |  | 2++ |
| conclusion: automatic classification performed better than subjective classification | | | | | | | | | | | |
| Dings, J. P.  Verhamme, L.  Merkx, M. A.  Xi, T.  Meijer, G. J.  Maal, T. J.  2019 | experimental study | meassurements in images of five dry human skulls | | measurements of five dry human skulls. |  | measurement in the images | measurement a the skulls | comparison of the accuracy of the measurements. | All radiologic measurements showed a significant overestimation of the bony dimensions, reaching more than the used voxel sizes of 0.3 mm for CBCT and 0.5 mm for MDCT. |  | 2++ |
| conclusion: Both CBCT images and MDCT images showed a highly consistent submillimeter overestimation of the anatomical truth. | | | | | | | | | | | |
| The 2007 Recommendations of the International Commission on Radiological Protection. ICRP publication 103. Annals of the ICRP. 2007  doi: 10.1016/j.icrp.2007.10.003. | guideline |  | |  |  |  |  | guidance on the control of exposure from radiation sources | They reinforce the principle of optimisation of protection, which should be applicable in a similar way to all exposure situations, subject to the following restrictions on individual doses and risks; dose and risk constraints for planned exposure situations, and reference levels for emergency and existing exposure situations. |  | 1+ |
| conclusion: recommendations to minimize radiation damage | | | | | | | | | | | |
| SEDENTEXCT project. RADIATION PROTECTION: CONE BEAM CT FOR DENTAL AND MAXILLOFACIAL RADIOLOGY  2011 | guideline |  | |  |  |  |  | guidelines on CBCT for dental and maxillofacial radiology | To give recommendations to the use of the CBCT in dentistry and maxillofacial radiology concerning the radiation protection. |  | 1+ |
| conclusion: recommendations to optimize the use of the CBCT | | | | | | | | | | | |
| Jaju, Sushma P  2015 Dec  doi: 10.5624/isd.2015.45.4.263 | narrative review |  | |  |  |  |  | appropriate radiation dosing during CBCT to the benefit of both patients and dentists | recommendations to minimize radiation exposure. |  | 2+ |
| conclusion: Two decades after the introduction of CBCT, it is time to move from ALARA to ALADA | | | | | | | | | | | |
| Gargallo-Albiol, Jordi  Barootchi, Shayan  Salomo-Coll, Oscar  Wang, Hom-Lay  2019  doi: 10.1016/j.aanat.2019.04.005 | systematic review |  | |  |  | fully guided | free hand, half guided | accuracy of implants, pain, patient satisfaction | Flapless surgery is related to reduced pain, less analgesic consumption, less swelling, shorter chair-time, and reduced risk of hemorrhage while achieving greater patient satisfaction. |  | 1++ |
| Conclusion: The FG associated with flapless surgery and teeth/crown supported guides has demonstrated the highest accuracy, followed by the drilling and pilot HG surgery which may provide comparable results, while non-computer HG and FH implant placement provide the least accuracy. | | | | | | | | | | |  |
| Vercruyssen, M.  Coucke, W.  Naert, I.  Jacobs, R.  Teughels, W.  Quirynen, M.  2015  10.1111/clr.12460 | randomized controlled trial | sixty patient,  311 Implants ( 6 groups with 51-55 Implants each) | |  |  | guided implantation | non-guided implantation | accuracy of implant placement | The overall mean vertical deviation for the guided surgery groups was 0.9 mm _ 0.8 (range: 0.0–3.7) and 0.9 mm _ 0.6 (range: 0.0–2.9) in a horizontal direction. For the non-guided groups, this was 1.7 mm _ 1.3 (range: 0.0–6.4) and 2.1 mm _ 1.4 (range 0.0–8.5), respectively  (P < 0.05). |  | 1++ |
| conclusion: The most important inaccuracy with guided surgery is in vertical direction (depth). The inaccuracy in MD or BL direction is clearly less. For non-guided surgery, the inaccuracy is significantly higher | | | | | | | | | | |  |
| Du Toit, J.  Gluckman, H.  Gamil, R.  Renton, T.  2015  DOI:10.1563/aaid-joi-D-14-00022 | case report and narrative review |  | |  |  |  |  | reasons for inferior alveolar nerve injury in dental implantology | recomondations when to use a CBCT in dental implantology |  | 2+ |
| conclusion: Panoramic imaging modalities remain the standard for planning assessment in implant dentistry, and CBCT imaging has a significant role for presurgical implant site assessment, implant placement, and follow-up radiographic assessment for specific cases. | | | | | | | | | | | |
| Ibrahim, N.  Parsa, A.  Hassan, B.  van der Stelt, P.  Aartman, I. H.  Wismeijer, D.  2014  DOI:10.1111/clr.12163 | experimental study | 24 mandibular cadavers | | 24 mandibular cadavers |  | cbct | micro CT | trabecular bone microstructural | Intra class correlation coefficients (ICC) showed a high intra-observer reliability (0.996) in all parameters for both systems. |  | 2+ |
| conclusion: Cone-beam CT datasets can be used to evaluate trabecular bone microstructure at dental implant sites. The accuracy for measuring Tb.N was the best followed by Tb.Th and Tb.Sp. | | | | | | | | | | | |
| Pauwels, R.  Sessirisombat, S.  Panmekiate, S.  2017  DOI: 10.11607/jomi.6210 | experimental study | 21 madibular cadavers | | 21 mandibular cadavers |  | cbct |  | preimplant CBCT scans: bone surface, bone volume, fractal dimension, connectivity, trabecular thickness and spacing, and skeleton analysis | The overall correlation was low to medium (|R| = 0.002—0.723). For the bone around the entire implant site, the highest correlation with ISQ was found for skeleton analysis and trabecular thickness. |  | 2+ |
| conclusion: bone structure parameters can have a predictive value in terms of primary implant stability, they should be measured at specific regions surrounding a planned implant site, and can provide complementary information | | | | | | | | | | | |
| Wrzesien, Malgorzata  Olszewski, Jerzy  Comparative Study  Journal Article  Poland  2017  doi: 10.13075/ijomeh.1896.00960. | experimental study | high-sensitivity thermoluminescent detectors (TLD) in 18 anatomical points of the phantom | |  |  | panoramic radiography, cephalometric radiography and cone beam computed tomography (CBCT) |  | absorbed doses of the brain, spinal column, thyroid and eye lens | The maximum absorbed dose recorded during performed measurements corresponds to the point representing the brainstem and it is 10 mGy. The dose value recorded by the TLD placed in the thyroid during CBCT imaging in relation to the panoramic radiography differs by a factor of 13.5. |  | 2+ |
| conclusion: Cone beam computed tomography, in comparison with panoramic or cephalometric imaging technique, provides higher radiation doses to the patients. | | | | | | | | | | | |

modified from Guidelines International Network – Evidence Tables Working Group: englisches Original verfügbar unter: <http://www.g-i-n.net/document-store/working-groups-documents/etwg-documents/template-evidence-summary-intervention-studies>. (Stand: 20.07.2011)
